# Cocaine intake correlates with risk-taking behavior and affects estrous cycling in female Sprague–Dawley rats

**DOI:** 10.3389/fnbeh.2023.1293226

**Published:** 2023-10-27

**Authors:** Leah M. Truckenbrod, Emily M. Cooper, Alexa-Rae Wheeler, Caitlin A. Orsini

**Affiliations:** ^1^Institute for Neuroscience, The University of Texas at Austin, Austin, TX, United States; ^2^Department of Psychology, The University of Texas at Austin, Austin, TX, United States; ^3^Department of Neurology, The University of Texas at Austin, Austin, TX, United States; ^4^Waggoner Center for Alcohol and Addiction Research, The University of Texas at Austin, Austin, TX, United States

**Keywords:** risk taking, estrous cycle, sex differences, cocaine, self-administration

## Abstract

Navigating complex decisions and considering their relative risks and rewards is an important cognitive ability necessary for survival. However, use of and dependence on illicit drugs can result in long-lasting changes to this risk/reward calculus in individuals with substance use disorder. Recent work has shown that chronic exposure to cocaine causes long-lasting increases in risk taking in male and female rats, but there are still significant gaps in our understanding of the relationship between cocaine use and changes in risk taking. For example, it is unclear whether the magnitude of cocaine intake dictates the extent to which risk taking is altered. To address this, male and female Sprague–Dawley rats underwent cocaine (or sucrose) self-administration and, following a period of abstinence, were trained and tested in a rodent model of risky decision making. In this behavioral task, rats made discrete-trial choices between a lever associated with a small food reward (i.e., “safe” option) and a lever associated with a larger food reward accompanied by a variable risk of footshock delivery (i.e., “risky” option). Surprisingly, and in contrast to prior work in Long-Evans rats, there were no effects of cocaine self-administration on choice of the large, risky reward (i.e., risk taking) during abstinence in males or females. There was, however, a significant relationship between cocaine intake and risk taking in female rats, with greater intake associated with greater preference for the large, risky reward. Relative to their sucrose counterparts, female rats in the cocaine group also exhibited irregular estrous cycles, characterized by prolonged estrus and/or diestrus phases. Collectively, these data suggest that there may be strain differences in the effects of cocaine on risk taking and highlight the impact that chronic cocaine exposure has on hormonal cyclicity in females. Future work will focus on understanding the neural mechanisms underlying cocaine’s intake-dependent effects on risk taking in females, and whether this is directly related to cocaine-induced alterations in neuroendocrine function.

## 1. Introduction

There are a range of psychiatric disorders that are characterized by forms of altered risk-taking behavior. Although certain disorders, such as eating disorders or generalized anxiety disorder, are characterized by extreme risk aversion, or avoidance of activities that may be associated with potential risk (Lorian and Grisham, 2011; Charpentier et al., 2017; Bernardoni et al., 2020), other psychiatric conditions are associated with increased risk-taking behavior. For example, individuals with substance use disorders (SUDs) display impaired risk-based decision making, undervaluing potential risks and overvaluing rewards (Chen et al., 2020), resulting in exaggerated risk-taking behavior. Not surprisingly, chronic drug use affects the neurobiological substrates that govern decision making, resulting in a disruption of an individual’s ability to properly evaluate choices (Rogers et al., 1999; Leland and Paulus, 2005; Wesley et al., 2011; Xiao et al., 2013). Neuroimaging studies have shown that individuals with SUDs exhibit differences in neural activity in key brain regions involved in risk/reward evaluation, such as the anterior cingulate cortex, orbitofrontal cortex, striatum, and amygdala (Bolla et al., 2003, 2004; Ersche et al., 2005; Bjork et al., 2008; Acheson et al., 2009). It is therefore necessary to investigate how neurobiological mechanisms of decision making may be disrupted following exposure to drugs of abuse.

The use of rodent models of risk-based decision making and intravenous cocaine self-administration has provided the opportunity to study the causal relationship between cocaine exposure and risk-taking behavior. Corroborating the alterations in decision making in humans with cocaine use disorder (Chen et al., 2020), self-administration of cocaine leads to long-lasting increases in the choice of larger, riskier options (Mitchell et al., 2014a; Blaes et al., 2022). Interestingly, studies using individuals with SUDs have reported that severity of drug use predicts the magnitude of certain cognitive deficits, with greater drug intake associated with worse cognitive performance (Verdejo-Garcia et al., 2006; Fernandez-Serrano et al., 2012). Consistent with these findings, others have reported similar intake-dependent effects of cocaine on changes in impulsive choice in rodents (Broos et al., 2012; Mitchell et al., 2014b). In particular, cocaine-induced increases in impulsive choice were only observed in rats that self-administered no less than 30 mg/kg of cocaine. It is unknown, however, whether there is also a threshold for cocaine’s effects on risk taking. If there is such a threshold, it could indicate that these individuals may be at greater risk for relapse than those who do not exhibit cocaine-induced changes in risk taking. Consequently, one goal of the current study was to extend these prior findings on the relationship between cocaine use and impulsive choice to determine whether there are intake-dependent effects of cocaine on risk-based decision making in male and female rats.

Another goal of the current study was to determine whether chronic cocaine exposure disrupts estrous cyclicity in females in parallel with cocaine’s effect on risk taking. Intriguingly, previous work has shown that ovariectomies increase risk taking (Orsini et al., 2021), mimicking the effects of chronic cocaine administration (Blaes et al., 2022). Hence, one possible explanation for the cocaine-induced increase in risk taking is that hormonal regulation of female risk-taking behavior has become compromised by chronic exposure to cocaine. Support for this contention comes from studies in non-human primates wherein self-administration of cocaine led to abnormalities in their menstrual cycle, and this disruption persisted during cocaine withdrawal (Mello et al., 1997). Additionally, passive cocaine exposure in rats similarly interrupts estrous cyclicity and decreases rates of ovulation (King et al., 1990, 1993). Notably, studies have found that the degree of cycle disruption and the ability to return to normal cycling following cocaine exposure occurred in a dose-dependent fashion (King et al., 1993). This suggests that in addition to intake-dependent effects on risk taking, cocaine intake may dictate the extent to which estrous cyclicity is disrupted. Finally, human female cocaine users experience amenorrhea and anovulation, although interpretation of these data is challenging due to confounding variables, such as poor nutrition and polysubstance use (Mello and Mendelson, 1997). Collectively, these findings provide evidence that chronic exposure to cocaine leads to long-lasting disruptions in normal hormonal cyclicity. As a first step in determining whether cocaine-induced changes in risk taking may be related to alterations in hormonal regulation of risk taking, we examined whether, in addition to altering cyclicity during cocaine self-administration, chronic cocaine use disrupted estrous cyclicity during the period of time that cocaine-induced increases in risk taking have been reported (Blaes et al., 2022).

**Figure 1:**
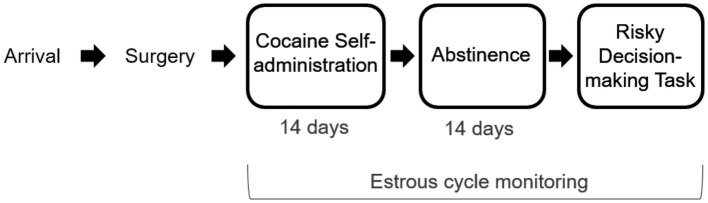
Experimental Timeline. Rats arrived and were allowed to acclimate to the facility before undergoing jugular catheter surgery. Following surgical recovery, rats were trained to self-administer cocaine under short-access conditions (2-h sessions), after which they underwent 14 days of long-access (6 h) self-administration sessions. A control group of rats was allowed to self-administer sucrose. Following two weeks of abstinence, rats were trained and tested on the Risky Decision-making Task until stable behavior emerged. Female rats’ estrous cycles were monitored during all phases of the experiment.

In this study, male and female rats underwent cocaine self-administration and, following a period of abstinence, were trained and tested in a rodent model of risk-based decision making (Figure 1). Intake-dependent effects of cocaine on risk taking were examined, as was the impact of cocaine on estrous cyclicity in females. We hypothesized that, similar to studies showing intake-dependent effects of cocaine on impulsive choice (Broos et al., 2012; Mitchell et al., 2014b), the magnitude of cocaine intake would determine the magnitude of the change in risk taking. In addition, we hypothesized that chronic cocaine self-administration would disrupt normal hormonal cyclicity in female rats. The findings of current study extend our understanding of the relationship between chronic cocaine use and changes in risk taking and begin to reveal potential biological mechanisms that may contribute to these cognitive changes in females.

## 2. Materials and methods

### 2.1. Subjects

Male and female Sprague–Dawley rats (*n* = 38, Envigo, Houston, TX) were used in the current experiments. Two separate cohorts (*Cohort 1*: male *n* = 8, female *n* = 10; *Cohort 2*: male n = 10, female n = 10) underwent the same behavioral procedures, as depicted in the experimental timeline in Figure 1. Rats were individually housed and maintained on a 12-h reverse light/dark cycle (lights off 0800 h; lights on 2000 h) to ensure testing was performed during the animals’ dark phase. Rats had free access to water throughout the experiment. During self-administration procedures, animals were mildly food restricted, receiving ~15 g of food each day (soy-free; Envigo Teklad Irradiated Global 19% Protein Extruded Rodent Diet, #2919), as mild food restriction can facilitate cocaine self-administration (Carroll et al., 1979). During behavioral testing in the Risky Decision-making Task, rats were further food restricted to 85% of their free-feeding weight, with the target weight increased by 5 g per week to account for growth. Procedures were approved by The University of Texas at Austin Institutional Animal Care and Use Committee and adhered to guidelines of the National Institutes of Health.

### 2.2. Apparatus

Separate behavioral chambers were used for testing in the Risky Decision-making Task (RDT) and cocaine self-administration. Both sets of plexiglass operant chambers had identical dimensions (Coulbourn Instruments, 31.5 × 31.5 × 26.2 cm) and were housed within a larger sound-attenuating cabinet with insulation foam (Coulbourn Instruments, 79.5 × 54 × 51.5 cm). The floors of each chamber consisted of stainless-steel rods through which scrambled footshocks were delivered via a shock generator (Coulbourn Instruments; note that shock was only delivered during the RDT). All operant chambers were interfaced with a computer running Graphic State 4 software (Coulbourn Instruments) that concurrently controlled task events and collected behavioral data.

#### 2.2.1. Self-administration

Each of the nine operant chambers was outfitted with two nosepokes capable of illumination. Nosepokes were located 10 cm from the floor of the chamber and flanked the left and right sides of a centrally located liquid reservoir trough. Situated 2 cm from the floor of the chamber, the reservoir trough contained a 1.12 W light bulb for illumination, a photobeam to register nosepoke entries, and a metal dipper that provided a 20% sucrose solution as a reward. Cocaine was delivered intravenously via a 20 mL syringe mounted on an infusion pump located in each chamber (Coulbourn Instruments). Each rat’s venous access port was connected to a tether system (Instech Laboratories) that consisted of spring-encased PE50 tubing that ran from the port implanted between the rat’s scapulae to a swivel mounted on the ceiling of the operant chamber and then to a syringe containing cocaine mounted on an infusion pump. Finally, each sound-attenuating chamber was equipped with a 1.12 W red-tinted house light mounted on the back of the chamber, along with a tone generator and speaker.

#### 2.2.2. Risky Decision-making Task

Testing in the decision-making task was conducted in nine operant chambers distinct from those used for self-administration. Each operant chamber contained a food trough, located 2 cm above the chamber floor and in the center of the front wall of the chamber. Food pellets (Test Diet, Lab Supply, 5TUL, Northlake, TX) were delivered from a food hopper positioned outside the test chamber into the food trough. Food troughs were outfitted with a 1.12 W light and photosensors to detect entries into the trough. Retractable levers were located on both sides of the food trough and 11 cm above the floor of the chamber. Locomotor activity was monitored and recorded using an activity monitor mounted on the ceiling of each operant chamber. Lastly, another 1.12 W red-tinted light was affixed to the back of the sound-attenuating chamber, serving as the house light.

### 2.3. Surgical procedures

Irrespective of self-administration group (i.e., cocaine vs. sucrose), all rats underwent jugular catheter surgery. Rats were anesthetized with isoflurane gas (1–5% in O_2_) and administered subcutaneous meloxicam (2 mg/kg), buprenorphine (0.03 mg/kg), and saline (10 mL). Hair on the rat’s back and chest was clipped and the underlying skin was disinfected with chlorohexidine and alcohol. A small incision was made in the skin above the jugular vein and the fat was dissected away to isolate the jugular vein. After ligating the vein with 5–0 suture, a small cut was made in the vein through which a catheter (Instech Laboratories) was inserted. The catheter was threaded into the length of the vein and then secured in place with additional 5–0 sutures. The opposite end of the catheter was passed subcutaneously over the right shoulder and through an incision between the rat’s scapulae. The catheter was attached to a venous access port (Instech Laboratories), which was then implanted beneath the skin. The incision around the backport was closed with sutures, cleaned with sterile saline, and treated with Vetricyn. Antibiotics (cefazolin, 0.1 mL, 30 mg/kg) were administered intravenously, followed by a lock solution (heparinized glycerol solution, 40 U/mL heparin in 50:50 glycerol:0.9% sterile saline), and then an aluminum cap was placed on the backport to protect it from debris. Catheters were flushed daily for 6 days with sterile saline (0.1 mL) and antibiotics (cefazolin; 0.1 mL; 30 mg/kg) and locked with the heparinized glycerol solution. Rats were given 7 days to recover from surgery before self-administration began. Patency of the catheters was tested prior to the start of self-administration with an intravenous infusion of propofol (0.1 mL). Catheters were deemed patent if an infusion resulted in a rapid but transient loss of muscle tone.

### 2.4. Behavioral procedures

#### 2.4.1. Cocaine self-administration

Cocaine self-administration procedures were identical to those used previously (Orsini et al., 2018, 2020; Blaes et al., 2022). Rats were first trained to associate the delivery of a 20% sucrose solution with the presentation of a tone (75–80 dB). If a rat made at least 100 reservoir entries to obtain the sucrose reward in a 60-min session, the rat then progressed to nosepoke shaping in which a rat learned that a nosepoke into the illuminated nosepoke (“active” nosepoke) triggered the delivery of the sucrose reward. Nosepokes into the other nosepoke hole (“inactive” nosepoke) were recorded but did not result in reward delivery. Rats were required to poke into the active hole at least 100 times in a 2-h session to progress to cocaine self-administration shaping. In this phase, rats were required to nosepoke into the active nosepoke to receive a 0.16 mL intravenous infusion of cocaine (1.0 mg/kg/infusion; dissolved in sterile saline) on an FR1 schedule. A nosepoke into the inactive nosepoke had no programmed consequences. Each infusion was accompanied by the presentation of the tone previously paired with delivery of sucrose. After a cocaine infusion, there was a 20-s timeout period in which both nosepokes were inactive. Rats were trained on this 2-h self-administration procedure for a minimum of 3 days and only progressed to long-access self-administration sessions (6 h/day) upon achieving 20 infusions in less than 2 h. Long-access self-administration lasted for 14 consecutive days. For all cocaine self-administration protocols (i.e., shaping and long-access), each cocaine rat was paired with a sucrose control rat that was tethered and run in the same operant box. During sucrose self-administration sessions, which always followed the daily cocaine self-administration session, the number of sucrose reinforcers (0.04 mL of 20% sucrose solution) each sucrose rat was allowed to self-administer was matched to the number of cocaine infusions their cocaine partner had received in the preceding cocaine self-administration session. This procedure therefore equated the number of reward opportunities across groups and controlled for aspects of self-administration unrelated to cocaine delivery (e.g., being tethered). After 14 days of long-access cocaine self-administration, all rats underwent a two-week abstinence period during which they remained in their homecage. Rats were only removed from their homecage for assessments of estrous cycle phase (or control manipulations in males).

#### 2.4.2. Risky Decision-making Task

##### 2.4.2.1. Shaping

Two weeks after the cessation of self-administration, rats began shaping procedures for the Risky Decision-making Task (RDT). Rats first learned to retrieve a single food pellet, delivered every 100 ± 40 s, from the food trough. To progress to the next phase of shaping, rats were required to make at least 100 trough entries within the 64-min session. In the next shaping procedure, one of the two levers (left or right side; counterbalanced across rats) was extended into the operant chamber for the entire 30-min session, and rats learned that a press on the extended lever resulted in the delivery of a food pellet. Once a rat made 50 lever presses in a single test session, they were shaped to press the opposite lever until they reached the same passing criterion. Upon reaching criterion on each lever, rats progressed to nosepoke shaping, in which they learned to nosepoke into the illuminated food trough to trigger extension of one of the two levers. A press within 10 s of lever extension resulted in the delivery of a food pellet. Rats were required to press each lever 30 times within 60 min to begin reward discrimination training.

##### 2.4.2.2. Reward discrimination

The Reward Discrimination (RD) task was used to train rats to make discrete-trial choices between two levers, one of which was associated with a small food reward (1 food pellet) and the other associated with a large food reward (2 food pellets). This task was 60-min in duration and consisted of 5 blocks of 18 trials. Each 40-s trial began with the illumination of the food trough, and a nosepoke into the food trough resulted in the extension of either one lever (forced choice trials) or both levers (free choice trials). If a rat failed to nosepoke within 10 s, the food trough and house lights were extinguished and the trial was scored as an omission. A press on one lever triggered the delivery of the small food reward, whereas a press on the opposite lever triggered the delivery of the large food reward. The identity of the lever (i.e., small vs. large) was counterbalanced across males and females and self-administration groups and was maintained throughout the duration of training in RD and the RDT. If a rat did not press a lever within 10 s of their extension, the house light was extinguished, the lever(s) were retracted and the trial was scored as an omission. The food trough light was terminated after the rat retrieved the food pellet or after 10 s elapsed since reward delivery, whichever occurred first. Each of the 5 blocks began with 8 forced choice trials wherein one lever was extended into the chamber (4 forced choice trials for each lever). The order of lever presentation during forced choice trials was randomized across trials. Subsequent to the forced choice trials, there were 10 free choice trials, during which both levers were extended into the operant chamber, and rats were allowed to freely choose between them. Unlike the RDT (see next section), each block of trials in RD was identical to one another. Despite this difference, training in RD served to inform the rats about the general task structure prior to training in the RDT. Rats were trained on the RD until they chose the large reward lever on at least 80% of the free choice trials for 3 consecutive days, at which point they began training on the RDT.

##### 2.4.2.3. Risky Decision-making Task

The RDT was identical in structure to RD with the exception that delivery of the large reward was accompanied by the possibility of mild footshock punishment. The probability of shock delivery increased across the 5 trial blocks in 25% increments, beginning at 0% and ending at 100%. Irrespective of the probability of footshock, the large reward was always delivered upon a press on the large, “risky” lever. The 8 forced choice trials that preceded the 10 free choice trials informed the rats of the punishment contingency in effect for that block. The probability of footshock delivery during forced choice trials was dependent across the four trials for the large lever. For example, in the 25% trial block, one and only one press on the large lever resulted in footshock delivery. In contrast, in the 75% trial block, 3 out of the 4 trials resulted in footshock delivery. Unlike forced choice trials, the probability of footshock delivery on a free choice trial was not dependent on the outcome of prior free choice trials. As in RD, a press on the small lever still resulted in the delivery of one food pellet with no other consequences (i.e., small, “safe” lever). For Cohort 1, shock intensities were 0.20 mA for males and 0.15 mA for females for the duration of testing in the RDT. For Cohort 2, shocks were initially 0.20 mA for males and 0.15 mA for females; at these shock intensities, however, rats in both self-administration groups were at the ceiling (i.e., no evidence of discounting). Consequently, shock intensities were adjusted upward to 0.225 mA for males and 0.175 mA for females. Rats were trained in the RDT until stable performance was reached, as defined in the *Data analysis* section.

### 2.5. Drugs

Cocaine HCl (NIDA Drug Supply Program) was dissolved in 0.9% sterile saline and administered intravenously at a dose of 1.0 mg/kg/infusion. Due to body weight differences between males and females, the cocaine solution was prepared separately for each sex.

### 2.6. Estrous cycle monitoring

Female animals’ estrous cycles were monitored throughout behavioral testing using vaginal lavage and cellular visualization procedures that have been previously described (Marcondes et al., 2002; McLean et al., 2012; Cora et al., 2015; Orsini et al., 2016). In Cohort 1, animals were only monitored for estrous cycle phase during testing in the decision-making task, while animals in Cohort 2 were tested across all experimental phases. Lavages occurred each day after behavioral testing and consisted of pipetting ~100uL of sterile saline into the animal’s vagina using a trumpeting technique. Samples were placed on glass microscope slides and viewed under a light microscope (10X). Phases of the estrous cycle (proestrus, estrus, metestrus, or diestrus) were determined by the relative presence of leukocytes, epithelial, and cornified cells in the sample. Male animals underwent penis palpation at the same time following daily behavioral testing to equate handling procedures across both sexes.

### 2.7. Data analysis

**Figure 2:**
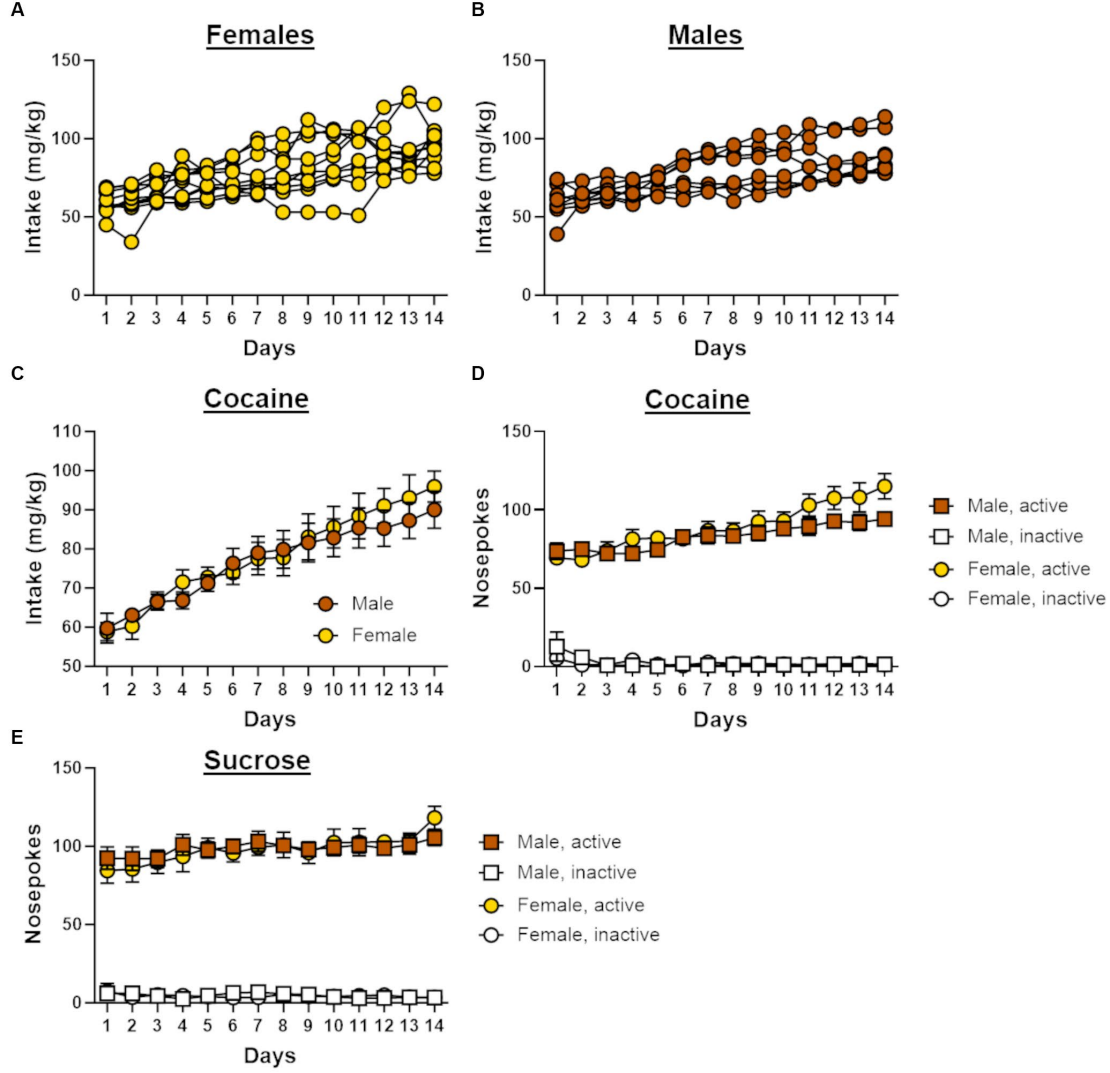
Self-administration behavior in male and female rats. **(A)** Individual cocaine intake for female rats (*n* = 10) across 14 days of self-administration. **(B)** Individual cocaine intake for male rats (*n* = 8) across 14 days of self-administration. **(C)** Cocaine intake increased across the 14 days of self-administration in both males and females. **(D)** Males and females in the cocaine group demonstrated a preference for the active nosepoke over the inactive nosepoke during self-administration. **(E)** Males and females in the sucrose group demonstrated a preference for the active nosepoke over the inactive nosepoke during self-administration. Data in C-E are represented as mean ± standard error of the mean (SEM). Absence of error bars (e.g., inactive nosepokes) is due to the size of the SEM being smaller than the point displayed.

Graphic State 4 data files were analyzed using custom Graphic State 4 analysis templates and exported and processed with Microsoft Excel analysis templates. Statistical analyses were performed using IBM SPSS Statistics 27, and figures were generated in GraphPad Prism 9. The primary dependent variables for cocaine self-administration were cocaine intake (mg/kg) and the number of “active” and “inactive” nosepokes. A repeated-measures analysis of variance (ANOVA) was used to analyze the change in cocaine intake across the two weeks of self-administration (day as a within-subjects factor) and to compare cocaine intake between sexes (sex as a between-subjects factor). To confirm a preference for the “active” nosepoke over the “inactive” nosepoke, another repeated-measures ANOVA was conducted, with nosepoke identity (“active” vs. “inactive”) and day included as the within-subjects factors and sex as the between-subjects factor. For correlational analyses, risk taking was averaged across blocks 2 through 5 (i.e., only trial blocks in which risk of punishment was present) of the RDT and cocaine intake was averaged across days 6–14. The selection of days of self-administration to include in this variable was chosen because the pattern of cocaine intake appeared to become more variable across rats on day 6 (suggesting possible individual differences in intake) and persisted in such a way until the end of self-administration (see Figures 2A,B). Pearson’s correlational analyses were then used to assess the relationship between risk taking and cocaine intake in males and females. Relationships between cocaine intake and performance on the RDT were also evaluated by dividing rats into “high cocaine intake” and “low cocaine intake” groups using a median split based on mean cocaine intake across days 6–14. A repeated-measures ANOVA was then used to determine whether risk taking differed between these intake groups, with trial block included as a within-subjects factor and intake group and sex included as between-subjects factors.

For the RDT, the primary behavioral measure was the percent of free choice trials in each block on which rats chose the large, “risky” lever. Stable choice performance on the RDT was determined by performing a repeated-measures ANOVA on free choice trials across a sliding window of three consecutive days of testing, with both day and trial block (i.e., risk of punishment) as within-subjects factors and sex and self-administration group as between-subjects factors. Behavior was deemed stable if there was a main effect of trial block, but an absence of both a main effect of day and a significant interaction between day and trial block in both sexes and self-administration groups. Choice of the large, risky reward for each trial block was then averaged across the three days of stable behavior and compared between self-administration groups and sexes using a repeated-measures ANOVA (group and sex included as between-subjects factors). Latencies to press levers during forced choice trials were calculated and subjected to a repeated-measures ANOVA, with lever identity (i.e., small, safe lever vs. large, risky lever) and trial block as the within-subjects factors and sex and self-administration group as the between-subjects factors. The percentage of omitted free choice trials was analyzed using a two-factor ANOVA, with sex and self-administration group as between-subjects factors. Similarly, baseline locomotor activity and locomotor activity during shock delivery were each analyzed with two-factor ANOVAs, with sex and self-administration group as between-subjects factors. If parent ANOVAs yielded significant main effects or interactions, additional ANOVAs or independent *t*-tests were conducted to identify the source of the significance. For all analyses, *p* ≤ 0.05 was considered statistically significant. If post-hoc analyses were conducted, Bonferroni-corrected *p*-values were used to determine if effects were statistically significant.

Estrous cycle phases were quantified and compared between cocaine and sucrose groups across the various phases of the experiment (self-administration, abstinence, and RDT testing), each of which lasted approximately 2–3 weeks. The criteria for a regular estrous cycle were: 1. a cycle duration of 4–5 days, and 2. evidence of a transition from proestrus/estrus to metestrus/diestrus across this 4-5-day duration (King et al., 1990) with estrus and diestrus phases lasting no longer than 3 and 4 consecutive days, respectively (Goldman et al., 2007). Using these guidelines, the number of regular estrous cycles in each experimental phase was averaged across rats in each self-administration group. This dependent variable was then compared between self-administration groups for each timepoint using an independent samples *t*-test; a repeated measures ANOVA was not used as there were uneven numbers of subjects across the different timepoints.

## 3. Results

### 3.1. Cocaine self-administration

Both male (n = 8) and female (n = 10) rats displayed similar patterns of escalation of cocaine intake across the 14 days of self-administration, such that intake increased over the course of testing [sex, *F* (1, 16) = 0.10, *p* = 0.76; day, *F* (13, 208) = 35.63, *p* < 0.01; day X sex, *F* (13, 208) = 0.81, *p* = 0.65; Figure 2C]. Rats in the cocaine group displayed a preference for the active nosepoke over the inactive nosepoke [nosepoke, *F* (1, 16) = 919.62, *p* < 0.01; Figure 2D], and the number of these nosepokes increased across the 14 days of self-administration [day, *F* (13, 208) = 8.05, *p* < 0.01; nosepoke X day, *F* (13, 208) = 18.27, *p* < 0.01]. Although a significant interaction between day and sex [*F* (13, 208) = 1.79, *p* = 0.05] suggested that nosepoke behavior differed between males and females during self-administration, there was no main effect of sex [*F* (1, 16) = 1.42, *p* = 0.25] nor were there other significant interactions with sex [nosepoke X sex, *F* (1, 16) = 1.46, *p* = 0.25; nosepoke X day X sex, *F* (13, 208) = 1.37, *p* = 0.18]. A repeated-measures ANOVA was used to compare nosepoke preference between self-administration groups and revealed that rats in the sucrose group (females, n = 9; males, n = 8) initially displayed greater preference for the active nosepoke over the inactive nosepoke compared with rats in the cocaine group [group, *F* (1, 29) = 10.75, *p* < 0.01; nosepoke X group, *F* (1, 29) = 4.47, *p* = 0.04]. Over the course of self-administration, however, rats in the cocaine group increased the number of active nosepokes to a greater extent than sucrose rats [*F* (13, 377) = 2.04, *p* = 0.02] such that both groups displayed comparable preference for the active nosepoke by the end of self-administration. This difference in nosepoke behavior between the self-administration groups was observed in both male and female rats [sex, *F* (1, 29) = 0.06, *p* = 0.82; sex X group, *F* (1, 29) = 1.54, *p* = 0.22; nosepoke X sex X group, *F* (29) = 1.41, *p* = 0.24; nosepoke X day X sex X group, *F* (13, 377) = 0.37, *p* = 0.98].

### 3.2. Risky Decision-making Task (RDT)

**Figure 3:**
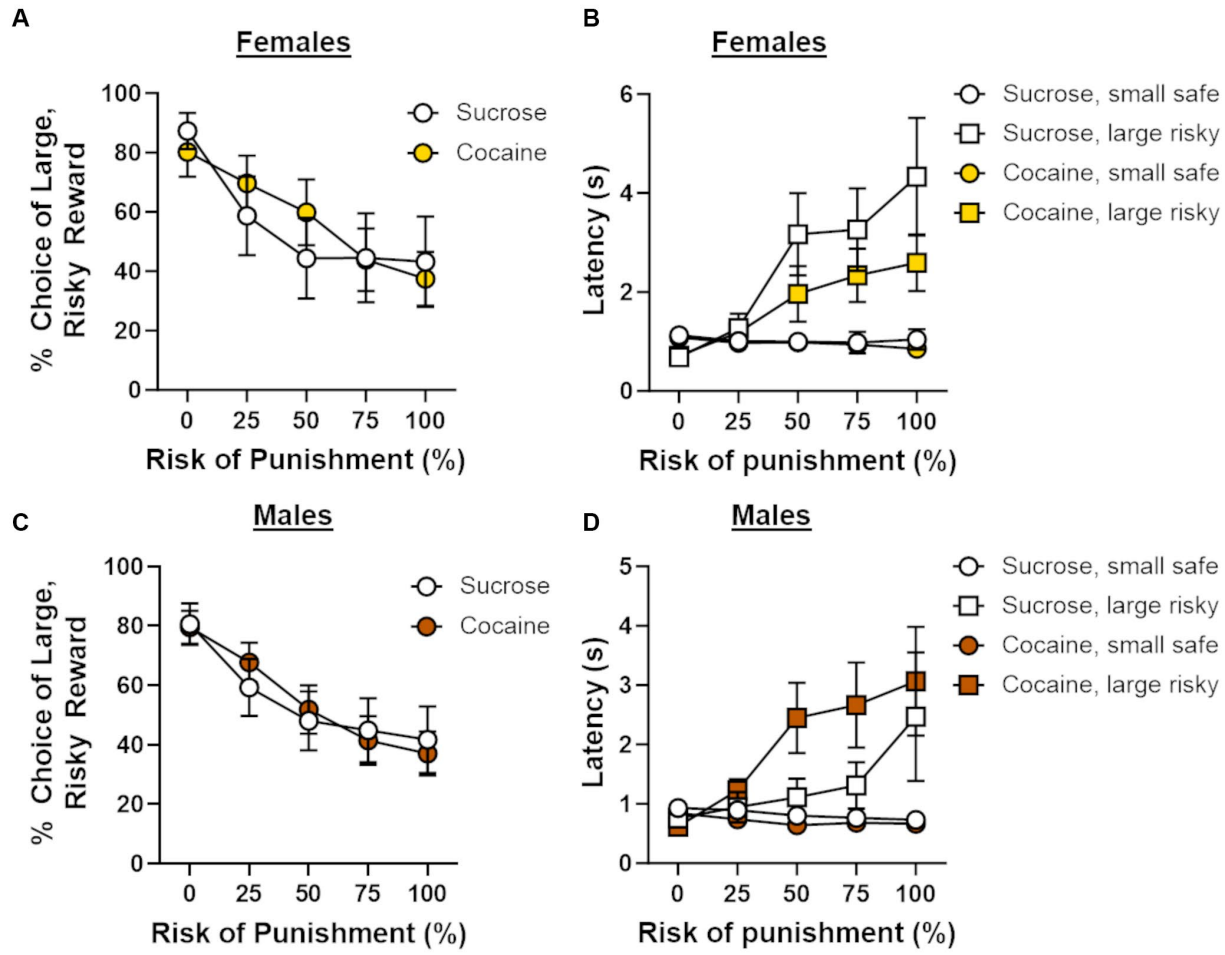
Effects of cocaine self-administration on risk-taking behavior. **(A)** There were no differences in choice of the large, risky reward between females that underwent cocaine (*n* = 10) or sucrose ((*n* = 9) self-administration. **(B)** Latencies to press the large, risky lever during forced choice trials increased as the risk of punishment increased in females in both the cocaine and sucrose self-administration groups. **(C)** There were no differences in choice of the large, risky reward between males that underwent cocaine (*n* = 8) or sucrose (*n* = 8) self-administration. **(D)** Latencies to press the large, risky lever during forced choice trials increased as the risk of punishment increased in males in both the cocaine and sucrose self-administration groups. Data are represented as mean ± standard error of the mean (SEM). Absence of error bars (e.g., latencies in the first block) is due to the size of the SEM being smaller than the point displayed.

Following cocaine self-administration, rats [cocaine group; male (n = 8), female (n = 10), sucrose group; male (n = 8), female (n = 9)] were trained on a Reward Discrimination task, after which they proceeded to testing on the RDT until behavioral stability emerged. There were no main effects of sex [*F* (1, 31) = 0.11, *p* = 0.75] or self-administration group [*F* (1, 31) = 0.63, *p* = 0.43] on the number of days required to reach passing criteria on the Reward Discrimination task, nor was there a sex X group interaction [*F* (1, 31) = 0.14, *p* = 0.71]. The average number of days to reach stability in the RDT differed between the self-administration groups by one day (30 days for sucrose group; 31 days for cocaine group). The lack of group differences in the number of days to train on RD and the RDT indicate that there were no overt differences in learning between the self-administration groups. Analysis of stable choice behavior in the RDT revealed an overall main effect of trial block [*F* (4, 124) = 35.33, *p* < 0.01], with choice of the large, risky lever (i.e., risk taking) decreasing as the risk of punishment increased. Because there was a main effect of trial block in all subsequent analyses (*p*s < 0.05), it will not be reported further. Risk taking did not differ between males and females [sex, *F* (1, 31) = 0.15, *p* = 0.71; sex X trial block, *F* (4, 124) = 0.22, *p* = 0.93], and, surprisingly, did not differ between self-administration groups [group, *F* (1, 31) = 0.14, *p* = 0.71; group X trial block, *F* (4, 124) = 0.65, *p* = 0.63; sex X group X trial block, *F* (4, 124) = 1.15, *p* = 0.34; Figures 3A,C]. A four-factor repeated-measures ANOVA (lever identity X trial block X sex X group) was used to compare latencies to press levers during forced choice trials between self-administration groups. Consistent with previous studies (Orsini et al., 2016, 2018), latencies to press the large, risky lever were significantly longer than those to press the small, safe lever [*F* (1, 29) = 15.02, *p* < 0.01] and increased as the risk of punishment increased [*F* (4, 116) = 24.16, *p* < 0.01; Figures 3B,D]. Latencies to press either lever did not differ by sex [sex, *F* (1, 29) = 2.74, *p* = 0.11; lever identity X sex, *F* (1, 29) = 0.36, *p* = 0.56; lever identity X sex X trial block, *F* (4, 116) = 1.53, *p* = 0.20] or self-administration group [group, *F* (1, 29) = 0.002, *p* = 0.96; lever identity X group, *F* (1, 29) = 0.23, *p* = 0.63; lever identity X group X trial block, *F* (4, 116) = 0.14, *p* = 0.97]. Although there was no interaction between lever identity, sex and group [*F* (1, 29) = 2.70, *p* = 0.11], there was a significant lever identity X sex X group X trial block interaction [*F* (4, 116) = 3.32, *p* = 0.01], suggesting that changes in latencies to press the small safe lever vs. the large, risky lever over the trial blocks differed between males and females and self-administration groups. To determine the source of this four-way interaction, latencies to press the small safe lever vs. the large risky lever were compared separately for females (Figure 3B) and males (Figure 3D) within each self-administration group. These analyses, however, were not particularly revealing, as the lever identity X trial block remained significant within each group (*p*s ≤ 0.01). Based on these results, it is unlikely that latencies to press levers differed reliably in a sex and group-dependent manner.

Additional analyses were conducted on ancillary behavioral measures (Table 1). Although females exhibited greater overall locomotor activity compared with males [*F* (1, 31) = 7.46, *p* = 0.01], locomotor activity did not differ between self-administration groups [group, *F* (1, 31) = 0.34, *p* = 0.57; sex X group, *F* (1, 31) < 0.01, *p* = 0.97]. When locomotor activity during the delivery of footshock was analyzed, there was neither a main effect of sex [*F* (1, 29) = 1.49, *p* = 0.23] nor a main effect of group [*F* (1, 29) = 0.25, *p* = 0.62], but there was a significant group X sex interaction [*F* (1, 29) = 4.73, *p* = 0.04]. Post-hoc analyses revealed that, after correcting for multiple comparisons, locomotor activity during shock delivery did not differ between male and females in the cocaine group [*t* (16) = −0.73, *p* = 0.48] nor did it differ between males and females in the sucrose group [*t* (13) = 2.21, *p* = 0.05]. As mentioned earlier, two cohorts of rats were used in these experiments and were tested in the RDT using different shock intensities (cohort 1: males, 0.20 mA and females, 0.15 mA; cohort 2: males, 0.225 mA and females, 0.175 mA). To confirm that the necessity of a higher shock intensity in rats in cohort 2 was not related to the quantity of self-administered cocaine, Pearson’s correlations were conducted for each cohort to assess the relationship between mean cocaine intake across days 6 through 14 and locomotor activity during footshock delivery. In cohort 1, there was a significant positive correlation between mean cocaine intake and locomotor activity during the delivery of footshock in rats (collapsed across males and females; r = 0.83, *p* < 0.06). When these analyses were performed separately in males and females, there were no significant associations between intake and locomotor activity in males (r = 0.89, *p* = 0.11) or females (r = 0.83, *p* = 0.83). In cohort 2, there was no significant relationship between cocaine intake and locomotor activity when the data were collapsed across sex (r = −0.54, *p* = 0.17). Similarly, when these analyses were conducted for each sex separately, there were no significant correlations between intake and locomotor activity in males (r = −0.92, *p* = 0.08) or females (r = −0.89, *p* = 0.09). Hence, the necessity of a higher shock intensity in cohort 2 to promote discounting of the large, risky reward does not appear to be related to the quantity of cocaine that was self-administered. Finally, the percentage of omitted free choice trials did not differ between males and females [*F* (1, 29) = 1.80, *p* = 0.19] or between self-administration groups [group, *F* (1, 29) = 0.88, *p* = 0.35; sex X group, *F* (1, 29) = 0.77, *p* = 0.39].

**Table 1:** Mean (± standard error of the mean) locomotor activity and omissions on the RDT.

	Locomotor activity *(locomotor units/ITI)*	Shock reactivity *(locomotor units/shock)*	Omissions (% of trials)
*Cocaine Group*			
*Male*	21.52 (3.46)*	2.79 (0.53)	0.08 (0.08)
*Female*	13.34 (2.23)*	3.30 (0.46)	2.87 (2.01)
*Sucrose Group*			
*Male*	19.87 (4.59)*	4.22 (0.77)	0.00 (0.00)
*Female*	11.47 (1.58)*	2.40 (0.37)	0.96 (0.51)

*indicates main effect of sex.

ITI, intertrial interval.

RDT, Risky Decision-making Task.

### 3.3. Relationship between cocaine self-administration and subsequent performance in the RDT

**Figure 4:**
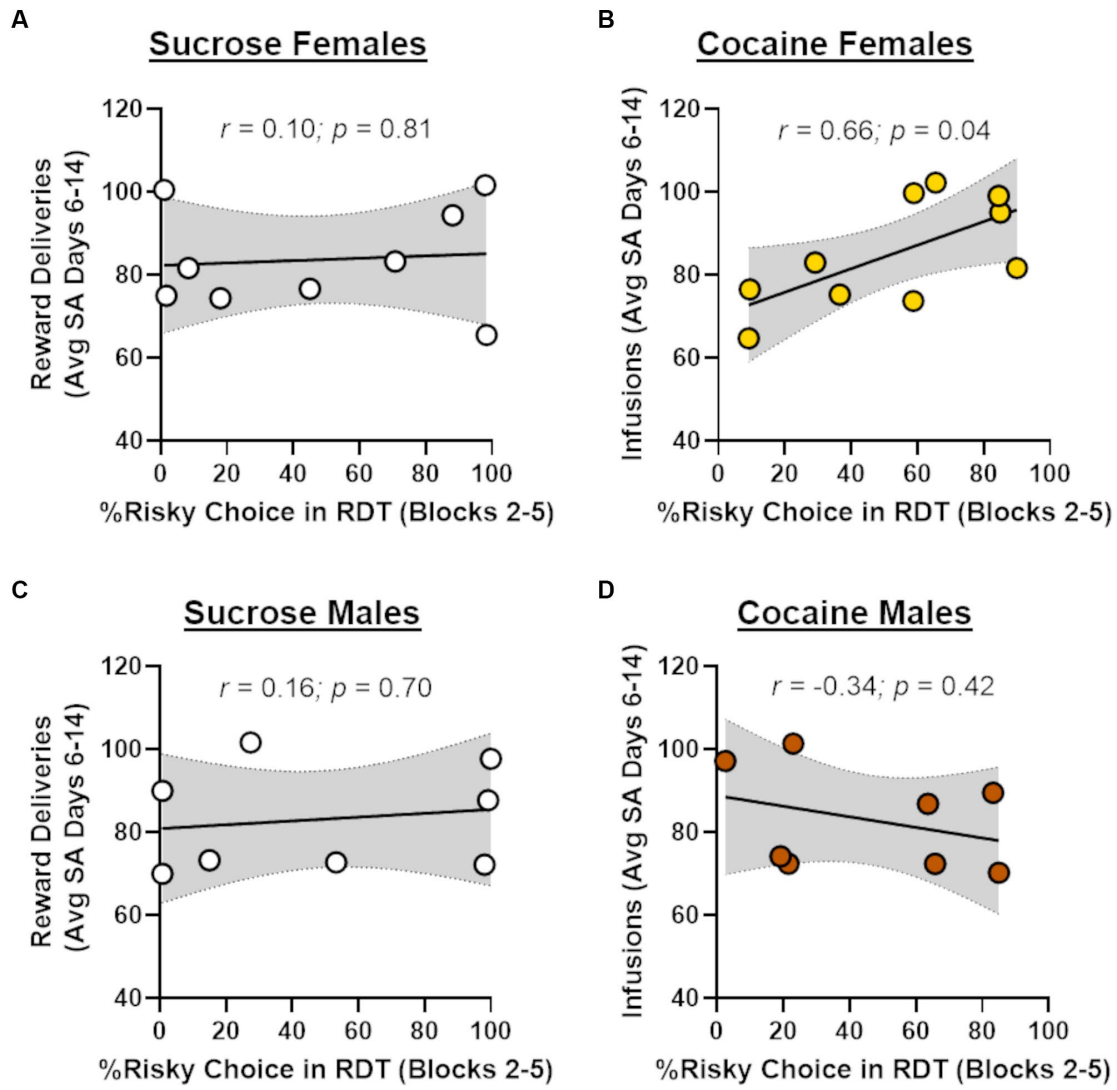
Relationship between cocaine intake and risk taking. **(A)** There was no significant relationship between choice of the large, risky reward and the number of sucrose reward deliveries in females that underwent sucrose self-administration (*n* = 9). **(B)** Greater choice of the large, risky reward was significantly correlated with a greater number of cocaine infusions (i.e., intake) in females that underwent cocaine self-administration (*n* = 10). **(C)** There was no significant relationship between choice of the large, risky reward and the number of sucrose reward deliveries in males that underwent sucrose self-administration (*n* = 8). **(D)** There was no significant relationship between choice of the large, risky reward and the number of cocaine infusions in males that underwent cocaine self-administration (*n* = 8).

**Figure 5:**
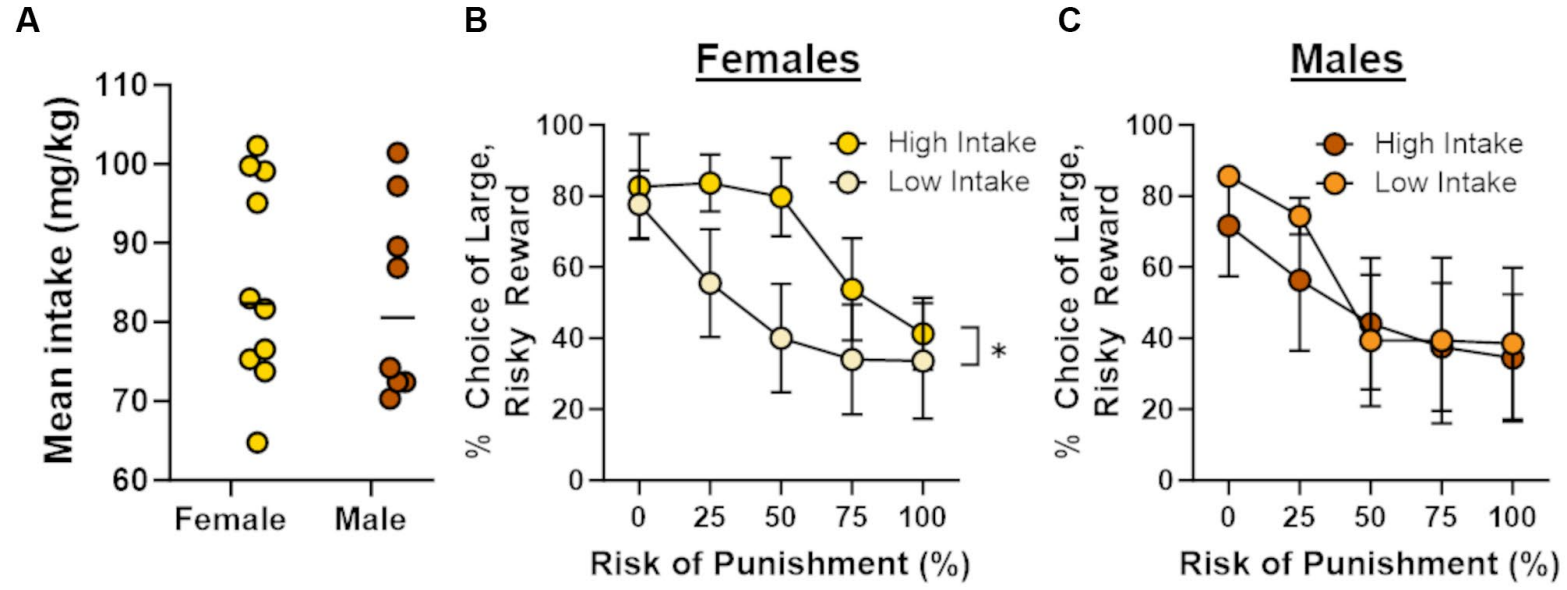
Intake-dependent effects of cocaine on risk taking. Rats were split into “high intake” and “low intake” groups. **(A)** Distribution of mean intake over days 6 through 14 of cocaine self-administration. Horizonal lines represent the median intake for each sex. **(B)** High intake females (*n* = 5) displayed a greater preference for the large, risky reward compared with low intake females (*n* = 5). **(C)** There was no difference between high intake (*n* = 4) and low intake (*n* = 4) males in their choice of the large, risky reward. Data are represented as mean ± standard error of the mean (SEM). Absence of error bars (e.g., males in block 1) is due to the size of the SEM being smaller than the point displayed. Asterisk indicates *p* < 0.05.

In light of recent work showing that cocaine self-administration increases in risk taking in males (Blaes et al., 2022), the absence of effects of cocaine on risk taking in males and females in the current study was unexpected. Previous studies, however, have demonstrated that the impact of cocaine on subsequent choice behavior can be dependent on cocaine intake during self-administration (Broos et al., 2012; Mitchell et al., 2014b). To explore this possibility, Pearson’s correlations were used to assess the relationship between the number of cocaine infusions during days 6–14 of self-administration and choice of the large risky reward in the RDT (averaged across blocks 2 through 5; Figure 4). Although there was no relationship between cocaine infusions and risk taking in male rats (*r* = −0.34, *p* = 0.42), there was a significant positive correlation between these behavioral measures in females (*r* = 0.66, *p* = 0.04). These data suggest that greater cocaine intake was associated with greater preference for the large, risky reward when females were tested in the RDT during abstinence. To determine whether this effect generalized to other rewards, similar correlations were performed in males and females in the sucrose group, using the number of sucrose deliveries instead of the number of cocaine infusions. There were no significant correlations between sucrose deliveries and performance in the RDT in males (*r* = 0.16, *p* = 0.70) or females (*r* = 0.10, *p* = 0.81), suggesting that the relationship between cocaine intake and risk taking in females was specific to cocaine. To further examine this relationship, rats in the cocaine group were divided into “high intake” and “low intake” groups using a median split of their average cocaine intake during days 6–14 of self-administration (Figure 5A). Although there was not a main effect of intake group in females [*F* (1, 8) = 1.33, *p* = 0.28], there was a significant interaction between intake group X trial block [*F* (4, 32) = 3.20, *p* = 0.03; Figure 5B]. Upon inspection of Figure 5B, this interaction appears to be driven by greater choice of the large, risky reward in the “high intake” group relative to the “low intake” group during the 25 and 50% risk blocks, although post-hoc comparisons between intake groups for each of these trial blocks were not statistically significant (*p* > 0.05). In contrast, there was no difference in subsequent risk taking between intake groups in males [intake group, *F* (1, 6) = 0.09, *p* = 0.77; intake group X trial block, *F* (4, 24) = 0.65, *p* = 0.63; Figure 5C].

### 3.4. Relationship between cocaine intake and estrous cycle disruption

**Figure 6:**
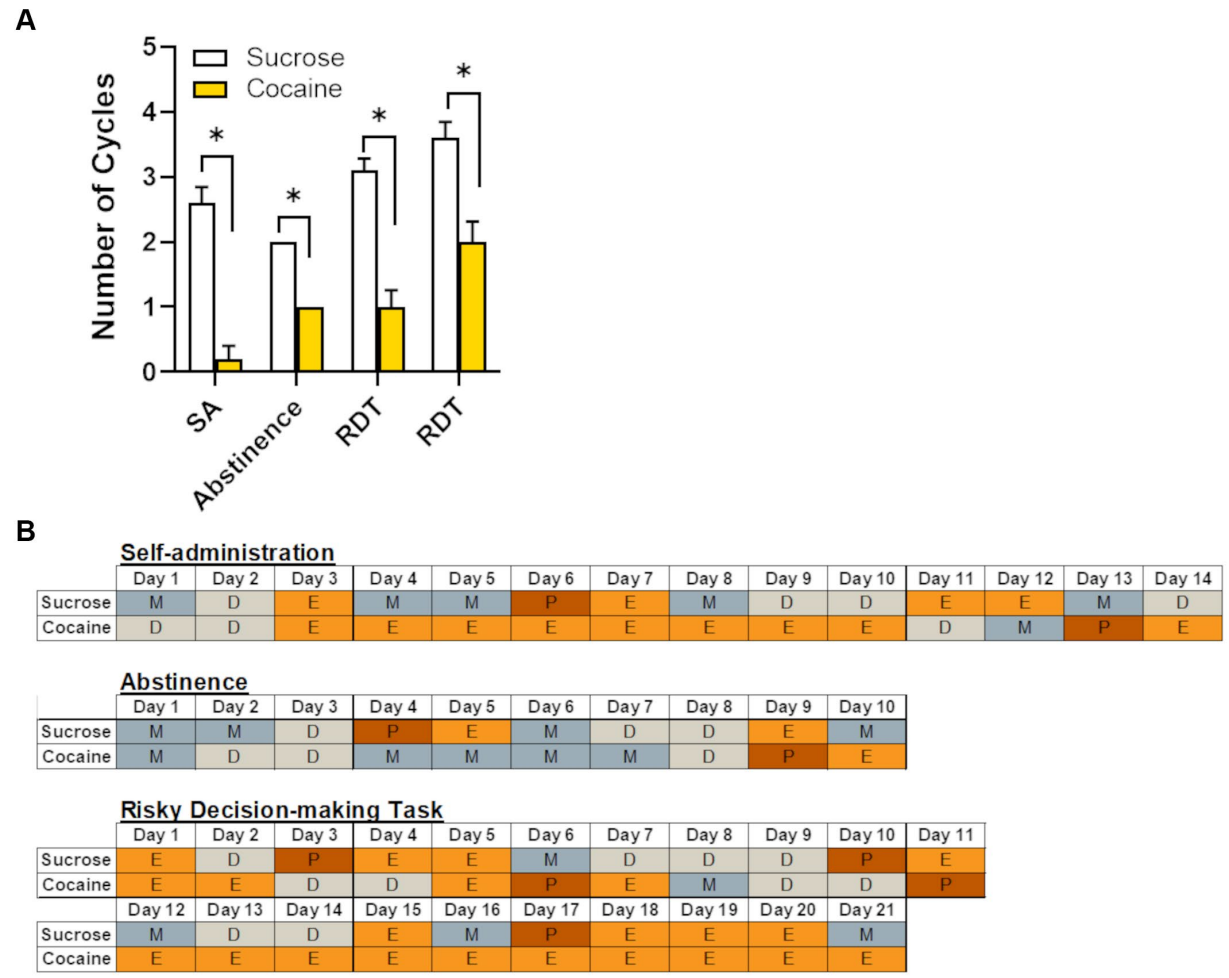
Effects of cocaine intake on female rats’ estrous cycles. **(A)** Relative to females in the sucrose group (*n* = 8), females in the cocaine group (*n* = 10) had fewer complete estrous cycles at each of the different experimental timepoints. Data are represented as mean ± standard error of the mean. The lack of error bars for each group during the abstinence phase is because rats in each group had an identical number of estrous cycles. Asterisks indicate *p* < 0.05. *SA,* self-administration; *RDT*, Risky Decision-making Task. **(B)** Representative estrous cycles of one female from the sucrose group and one female from the cocaine group during each experimental timepoint. *P*, proestrus; *E*, estrus; *M*, metestrus; *D*, diestrus.

Chronic cocaine has been shown to disrupt natural hormonal cycling in rodents, non-human primates and women who are currently using or have a history of using cocaine (King et al., 1990,^1993^; Mello et al., 1997; Mello and Mendelson, 1997). Consistent with these reports, female rats in the cocaine group displayed significantly fewer complete estrous cycles (as defined in the Methods) across the various time points of the experiment (i.e., self-administration, abstinence, and RDT testing; Figure 6). Although each of these windows of time were equivalent in length (approximately 2–3 weeks), the estrous cycle of the first cohort of rats was tracked only during testing in the RDT, unlike the second cohort in which the cycle was monitored throughout self-administration, abstinence and testing in the RDT. This resulted in an unequal number of subjects for each timepoint. Consequently, data was analyzed using an independent samples *t*-test (as opposed to a repeated-measures ANOVA), comparing the number of cycles between sucrose and cocaine self-administration groups at each timepoint. Relative to their sucrose counterparts, rats that self-administered cocaine had significantly fewer estrous cycles during self-administration [n = 5 per group; *t* (8) = −4.81, *p* < 0.01], abstinence [*n* = 5 per group; *t* (8) = −4.00, *p* < 0.01] and RDT testing [first three weeks: *n* = 10 per group; *t* (18) = −5.46, *p* < 0.01; second three weeks: *n* = 5 per group, *t* (8) = −4.00, *p* < 0.01]. Figure 6B provides representative examples of estrous cycles of females in the cocaine and sucrose group across each experimental timepoint. As evident in Figure 6B, estrous cycles in the females in the cocaine group were characterized by prolonged estrus or diestrus phases. In contrast, females in the sucrose group exhibited typical 4–5 day cycles, with transitions from high ovarian hormone states (proestrus/estrus) to low ovarian hormone states (metestrus/diestrus) within this time window.

Given this disruption in hormonal cyclicity in the cocaine group, regression analyses were performed to determine whether the magnitude of cocaine intake influenced the degree of cycle disruption. These analyses were limited to estrus cycle measurements during testing in the RDT, as there were insufficient data to perform these analyses on estrus cycle measurements during self-administration and the abstinence timepoints. Because the estrous cycle variable consisted of count data that are positively skewed (i.e., bounded by 0), a Poisson regression was used to test the effect of cocaine intake on the number of cycles in females in the cocaine group (n = 10). This regression used the number of estrous cycles during RDT testing as the dependent variable and the mean cocaine intake (across days 6–14) as the independent variable. The results of the Poisson regression are presented in Table 2. Although there were effects of self-administration group on the number of estrous cycles (consistent with Figure 6A), there was no statistically significant effect of mean cocaine intake of the number of estrous cycles during RDT testing in females that self-administered cocaine (*β* = 0.004, *p* = 0.87). It is important to note that there was limited variability in intake levels within this group, with all females self-administering similarly high quantities of cocaine. Altogether, these data indicate that chronic exposure to cocaine causes disruptions in estrous cyclicity, although this does not appear to scale with the magnitude of cocaine intake.

**Table 2:** Poisson regression estimation of estrus cycles in female cocaine rats.

	Coefficient estimate
Average Cocaine Intake	0.004
*S.E.*	*(0.026)*
*p*-value	*0.872*
Intercept	−0.352
*S.E.*	*(2.213)*
*p*-value	*0.873*
Likelihood Ratio $\chi^{2}$	0.03
*p*-value	0.872
Pseudo-R^2^	0.001

***, **, and * represent statistical significance at the 99, 95, and 90% level. S.E. = coefficient standard error.

## 4. Discussion

The aim of the current study was to extend our understanding of the effects of cocaine exposure on risk-taking behavior on the Risky Decision-making task (RDT), a model of risk-based decision making in rats. We have previously shown that exposure to cocaine, either via passive administration or intravenous self-administration, increased subsequent risk taking on the RDT (Blaes et al., 2022). Although we were sufficiently powered to determine that there were no sex differences in the effects of passive cocaine administration on risk taking in this previous study, we were underpowered to address sex differences in the effects of intravenous cocaine self-administration on risk taking. Consequently, in the current study, we sought to extend the findings of our previous work with sufficient numbers of males and females in each self-administration group (i.e., cocaine and sucrose). Surprisingly, we did not observe effects of cocaine self-administration on risk taking in males or females. There was, however, a significant positive correlation between cocaine intake and risk taking in females that self-administrated cocaine, suggesting that the magnitude of cocaine intake may dictate the severity of impairments in risk taking. Intriguingly, we also observed that, relative to females in the sucrose group, females in the cocaine group exhibited persistent disruptions of their estrous cycles, beginning during self-administration and lasting through testing on the RDT. These findings suggest that, in addition to altering cognitive function in females, cocaine may have a lasting effect on their hormonal cyclicity and reproductive health.

### 4.1. Cocaine intake and risk-taking preference

The lack of effects of cocaine self-administration on risk taking was unexpected and is incongruent with previous work in which cocaine exposure increased risk taking in males and females (Mitchell et al., 2014a; Blaes et al., 2022). Others have also shown that cocaine self-administration increases choice of risky options in males in a rodent version of the Iowa Gambling Task (Ferland and Winstanley, 2017). However, one significant difference between the current study and our previous work (Blaes et al., 2022) is that different strains of rats were used in each study: while Blaes et al. used Long-Evans rats, the current study used Sprague–Dawley rats. Recent evidence has shown that factors related to decision making and other reward-related behavior can differ between different outbred (Pitchers et al., 2015; Rivera-Garcia et al., 2020) and inbred rat strains (Suzuki et al., 1988, George and Goldberg, 1989, George et al., 1991, Guitart et al., 1993, Nestler et al., 1993, Nestler, 1994). For example, there are sex differences the propensity for Sprague–Dawley rats to attribute incentive salience to cues predictive of food rewards; such sex differences, however, do not exist in a heterogenous outbred rat strain (Pitchers et al., 2015). Further, there are striking sex differences in both fixed and dynamic properties of the dopaminergic system in Sprague–Dawley rats that are otherwise absent in Long-Evans rats (Rivera-Garcia et al., 2020). Consistent with these findings, others have reported strain differences in central monoamine levels (Scholl et al., 2010) and sensitivity of behavioral responses to dopamine receptor agonists (Swerdlow et al., 2000). It is therefore conceivable that the inconsistency between our current and previous findings is due to the use of Sprague–Dawley rats instead of Long-Evans rats. In our experience, recovery from surgery in Long-Evans rats can be challenging as they are more inclined to aggravate their incision sites. We therefore chose the Sprague–Dawley strain due to their docile nature, which we believed would improve surgical recovery. Although the use of a different strain did indeed improve recovery from surgery, it may have inadvertently impacted the ability to replicate our previous work. Hence, future efforts will focus on conducting the current experiment in Long-Evans male and female rats and then directly comparing the results between strains.

Despite the overall lack of effects of cocaine on risk taking, we did observe a significant relationship between cocaine intake and risk taking in females. Specifically, greater cocaine intake during self-administration was correlated with greater risk taking during abstinence in females. Further, when female rats were divided into high and low cocaine intake groups, rats in the high intake group chose the large, risky reward significantly more than those in the low cocaine intake group during subsequent RDT testing. These findings are consistent with prior work in rats showing that effects of cocaine on impulsive choice are also intake dependent (Broos et al., 2012; Mitchell et al., 2014b). Intake-dependent effects of cocaine on risk taking are also reminiscent of findings from studies of cocaine users in which the severity of an individual’s cognitive impairments scaled with the length of cocaine use (Verdejo-Garcia et al., 2006; Fernandez-Serrano et al., 2012). Collectively, these findings suggest that individual differences in cocaine intake (either magnitude of intake or duration of use) could potentially determine the degree of the impairments in decision making and cognition during abstinence. This, in turn, may confer greater vulnerability to relapse. It was, however, surprising that this relationship between cocaine intake and risk taking was only evident in females. Because intake-dependent effects of cocaine on impulsive choice have been observed in Long-Evans males (Mitchell et al., 2014b), it is possible that the use of Sprague–Dawley male rats precluded the ability to observe a similar relationship between cocaine intake and subsequent risk taking in males. A comparison between strains in both males and females is therefore necessary to better understand the reproducibility and specificity of this association.

### 4.2. Cocaine intake and estrous cycle

The relationship between hormonal state and the rewarding properties of cocaine is complex. Female rats display enhanced preference for cocaine-associated cues when the initial pairing of cues and cocaine occurs during the estrus phase of the estrous cycle (Calipari et al., 2017). Such hormone-mediated augmentation in the rewarding properties of cocaine is thought to be due to enhanced potency of cocaine in inhibiting dopamine clearance in the nucleus accumbens. These data in rodents corroborate findings in female cocaine users, who report a greater “high” from cocaine administration during the follicular phase of the menstrual cycle (the phase in which the level of ovarian hormones is high; Evans et al., 2002, Evans, 2007). Chronic cocaine exposure, however, can directly impact hormonal cyclicity. Women who chronically use cocaine report disruptions in their menstrual cycle, typically in the form of amenorrhea, or the cessation of menses for a prolonged period (Mello and Mendelson, 1997). Interpretation of results of studies in these women is complicated, however, by the fact that women are using cocaine in combination with other substances, such as opioids and alcohol. Studies in non-human primates, who experience hormonal cyclicity similar to women, show that cocaine self-administration does disrupt regular menstrual cycles (Mello et al., 1997), indicating that the perturbations in women’s menstrual cycles are at least in part due to cocaine and may in fact be compounded by the use of additional substances. Consistent with these findings, passive administration of cocaine in rats results in irregular estrous cycles (King et al., 1990, 1993). Our results extend this work by showing that intravenous cocaine self-administration leads to abnormal estrous cycles during the period of drug use and this acyclicity persists for at least 7–8 weeks after self-administration. Using methods previously employed to characterize changes in estrous cyclicity (Goldman et al., 2007), we observed that disruptions of the estrous cycle in rats that self-administered cocaine were characterized by prolonged periods of diestrus and/or estrus and an absence of proestrus. Although others have shown that the degree of cycle disruption is dependent on the dose of cocaine (King et al., 1993), there was no relationship between cocaine intake and the number of regular cycles during testing in the RDT in the current study. This lack of relationship, however, could be due to low sample sizes in the self-administration groups and insufficient data to perform these analyses at earlier timepoints (i.e., self-administration and abstinence). Rats also did not receive a wide range of cocaine doses, as in the King et al. study. Consequently, additional data are needed to more definitively address whether the severity of cycle disruption is related to the amount of cocaine self-administered. These limitations notwithstanding, the similarity of the effects of ovariectomies, which lead to the cessation of estrous cycling, and cocaine exposure, which induces cycle irregularity, on risk taking in the RDT suggests that alterations in hormonal regulation of risk taking may account for cocaine-induced changes in risk taking in females. One mechanism by which these changes could occur is through a disruption in dopaminergic modulation of risk taking. Indeed, the gonadal hormone estradiol regulates dopaminergic transmission in the striatum (Yoest et al., 2018, Quigley et al., 2021), and dopamine signaling within this same brain region is necessary for decision making involving risk of punishment (Mitchell et al., 2014a; Truckenbrod et al., 2023). Hence, changes in hormonal fluctuations as a consequence of cocaine exposure may alter striatal dopamine dynamics that contribute to balanced risk-taking behavior.

### 4.3. Limitations and future directions

One limitation of the current study is that rats were not trained in the RDT prior to self-administration, precluding the ability to compare risk taking in rats that self-administered cocaine to a drug-naïve baseline. This experimental design was chosen, however, based on our prior work in which we showed that the age at which cocaine self-administration occurs dictates the effects of cocaine on risk taking (Blaes et al., 2022). In Blaes et al., we showed that the effects of cocaine exposure on risk taking are only evident when this exposure occurs in young adulthood, and this effect is absent when exposure occurs in later adulthood. Because there is no drug-naïve baseline to which to compare risk taking after self-administration, we cannot rule out the possibility that the intake-dependent effects of cocaine on risk taking in females are due to slight impairments in learning rather than alterations in decision making. Arguing against this interpretation is the fact that there were no differences between self-administration groups on the number of days required to reach criteria on reward discrimination training. Moreover, rats in the sucrose and cocaine group required approximately the same number of days (30 vs. 31, respectively) to reach behavioral stability on the RDT. Given the similarity in these learning trajectories between groups, it is less likely that the intake-dependent effects of cocaine on risk taking in females are merely due to impairments in learning or the inability to discriminate between the levers.

Although we show that cocaine exposure disrupts estrous cyclicity in female rats during and after self-administration, a major caveat to this finding is that a baseline measure of estrous cyclicity prior to self-administration was not included. We were therefore limited to only using estrous cycles from rats in the sucrose control group as a comparison. Future experiments will include estrous cycle assessments prior to self-administration to provide a within-subjects comparison timepoint. In addition, blood serum will be collected at multiple timepoints to evaluate peripheral hormone levels (e.g., estradiol, progesterone, corticosterone) in males and females. Finally, even though cocaine did not affect risk taking in males, it is possible that, similar to females, it disrupted their endogenous hormonal cyclicity. In contrast to females in which estradiol and progesterone fluctuate over a 4–5 day period, testosterone fluctuates over a 24-h period in males, with elevations occurring early in their dark cycle (Tcholakian and Keating, 1978; Keating and Tcholakian, 1979). There is limited and mixed evidence for detrimental effects of cocaine on male reproductive health (Berul and Harclerode, 1989; Mello and Mendelson, 1997; Mendelson et al., 2003; Alves et al., 2014); future studies will therefore be designed to also assess the effects of cocaine on diurnal testosterone fluctuations (e.g., via blood collection and serum hormone analysis).

### 4.4. Conclusion

In summary, our results suggest that the magnitude of cocaine intake during cocaine use may determine the severity of subsequent impairments in decision making during abstinence, but only in females. Further, chronic cocaine exposure causes long-lasting disruptions in hormonal cyclicity in females. These findings provide foundational information for understanding the biological mechanisms by which decision making may become compromised following cocaine use. Moving forward, it will be crucial to continue to study the extent to which hormonal dysregulation of risk taking contributes to drug-induced changes in cognitive abilities, such as risk-based decision making.

## Data availability statement

The raw data supporting the conclusions of this article will be made available by the authors, without undue reservation.

## Ethics statement

The animal study was approved by Institutional Animal Care and Use Committee at UT Austin. The study was conducted in accordance with the local legislation and institutional requirements.

## Author contributions

LT: Data curation, Investigation, Methodology, Writing – original draft. EC: Data curation, Writing – review & editing. A-RW: Data curation, Methodology, Writing – review & editing. CO: Conceptualization, Formal analysis, Funding acquisition, Methodology, Project administration, Resources, Supervision, Writing – review & editing.
